# Molecular Mechanisms of Intercellular Rhizobial Infection: Novel Findings of an Ancient Process

**DOI:** 10.3389/fpls.2022.922982

**Published:** 2022-06-23

**Authors:** Johan Quilbé, Jesús Montiel, Jean-François Arrighi, Jens Stougaard

**Affiliations:** ^1^Department of Molecular Biology and Genetics, Aarhus University, Aarhus, Denmark; ^2^Centre for Genomic Sciences, National Autonomous University of Mexico (UNAM), Cuernavaca, Mexico; ^3^IRD, Plant Health Institute of Montpellier (PHIM), UMR IRD/SupAgro/INRAE/UM/CIRAD, Montpellier, France

**Keywords:** intercellular symbiosis, legumes, nodule, *Lotus*, *Aeschynomene*, *Arachis*, *Sesbania*

## Abstract

Establishment of the root-nodule symbiosis in legumes involves rhizobial infection of nodule primordia in the root cortex that is dependent on rhizobia crossing the root epidermal barrier. Two mechanisms have been described: either through root hair infection threads or through the intercellular passage of bacteria. Among the legume genera investigated, around 75% use root hair entry and around 25% the intercellular entry mode. Root-hair infection thread-mediated infection has been extensively studied in the model legumes *Medicago truncatula* and *Lotus japonicus*. In contrast, the molecular circuit recruited during intercellular infection, which is presumably an ancient and simpler pathway, remains poorly known. In recent years, important discoveries have been made to better understand the transcriptome response and the genetic components involved in legumes with obligate (*Aeschynomene* and *Arachis* spp.) and conditional (*Lotus* and *Sesbania* spp.) intercellular rhizobial infections. This review addresses these novel findings and briefly considers possible future research to shed light on the molecular players that orchestrate intercellular infection in legumes.

## Introduction

**Figure 1:**
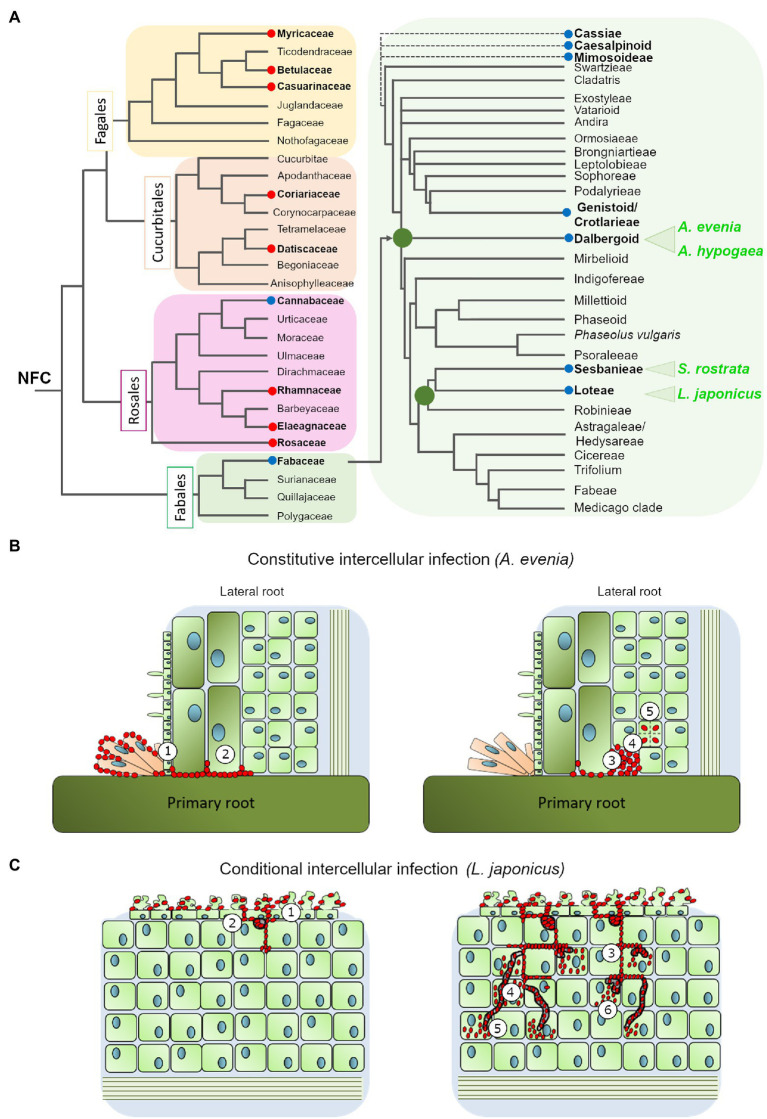
**(A)** Phylogenetic relationship of the nitrogen-fixing clade (based on Sun et al., 2016; Shen and Bisseling, 2020) with special emphasis in the Fabaceae family (modified from Sprent et al., 2017). The red and blue circles highlight the genera where intercellular colonization by Frankia or Rhizobia occurs, respectively. The four species discussed in this review are marked in green **(B)**. Intercellular infection mechanism in *Aeschynomene evenia*: the bradyrhizobium ORS278 intensively colonizes the axillary root hairs at the lateral root base and progresses between cells (1) to reach the cortex (2) where they could induce cell-collapse (3), before bacteria are finally internalized (4) and induce cell division (5). **(C)** Intercellular infection mechanism in *Lotus japonicus*: IRBG74 induce massive root hair curling and twisting, followed by intercellular infection of the root epidermis (1). In the cortical cells, IRBG74 is accumulated in infection pockets (2) and from these structures migrates to the nodule cells intercellularly (3) or through transcellular infection threads (4). The bacteria are released into the nodule cells from transcellular infection threads (5) or intercellular infection peg structures (6).

The root nodule symbiosis (RNS) has been widely studied in the model legumes *Lotus japonicus* and *Medicago truncatula*. In these species, rhizobia infect the roots *via* a root-hair infection thread (IT), after a chemical recognition that occurs in the rhizosphere. The molecular dialog involves the secretion of flavonoid root exudates that are perceived by the rhizobial partner. In response, the microsymbiont synthesizes and releases lipochito-oligosaccharides known as nodulation factors (NF), which are recognized by the NF receptors located at the root hair plasma membrane. This complex signal exchange leads to the formation of the nodule primordia by reactivation of cortical cell division. This structure gives rise to the root nodule, where the bacteria are released from the ITs into the nodule cells to become bacteroids, carrying out the conversion of atmospheric nitrogen into ammonia (Roy et al., 2020). Among the six subfamilies of the Fabaceae (Koenen et al., 2020), only the Caesalpinioideae (+Mimosoid clade) and Papilionoideae are considered to harbor nodulating genera and it is estimated that about one quarter of all legume genera employ a rhizobial invasion programme called intercellular infection (Sprent et al., 2017). This infection process is presumed to be a simpler mechanism than root-hair IT-mediated infection that involves many biological functions (Roy et al., 2020). Furthermore, since intercellular infection is found in different legume subfamilies (Sprent et al., 2017; Koenen et al., 2020), and beyond the legume family in the Parasponia-Bradyrhizobium symbiosis and in certain actinorhizal plants (Figure 1A), it is likely to be an ancient and fundamental mechanism (Huisman and Geurts, 2020). Intercellular infection comprises different modalities that have been described through histological analyses in several species (James et al., 1992; Subba-Rao et al., 1995; Ibañez et al., 2017). These modalities need to be characterized at a molecular level. Here, we review emerging knowledge of the molecular genetics of nodulation by intercellular infection, and discuss why exploring these mechanisms in legumes, where this process is either obligate or conditional, is complementary.

## Conditional Intercellular Infection

Certain legume species are remarkable because although they usually employ root hair ITs infection they can in certain conditions switch to the intercellular infection process. Such plasticity in the infection mode has repeatedly (although not exclusively) been observed within the robinoid subclade, in *Sesbania* spp. and *Lotus* spp. (Figure 1A; Ndoye et al., 1994; Cummings et al., 2009; Acosta-Jurado et al., 2016; Mitra et al., 2016; Liang et al., 2019). In these species, intercellular colonization either takes place under specific growth conditions or is observed with specific rhizobial partners (Ranga Rao, 1977; Karas et al., 2005). In the semi-aquatic legume *Sesbania rostrata*, root hair ITs are readily observed in aeroponic conditions, while lateral root base (LRB) nodulation is observed under flooded conditions, *via* a crack-entry mechanism in which *Azorhizobium caulinodans* enters through epidermal fissures of emerging adventitious roots (Capoen et al., 2010b). Similarly to *S. rostrata*, under flooded conditions the robinoid *Lotus uliginosus* is intercellularly colonized by *M. loti*, culminating in the formation of nitrogen-fixing nodules (James and Sprent, 1999). Another *Lotus* species, *L. burttii*, is an interesting model for studying rhizobial infection, since it can be nodulated by a large diversity of rhizobial species (Zarrabian et al., 2021). The evidence collected indicates that some of these associations occur intercellularly; for instance, the interactions with *Sinorhizobium fredii* HH103 (Acosta-Jurado et al., 2016) and *Rhizobium leguminosarum* biovar Norway (Liang et al., 2019), the latter leading to ineffective nodules. In the model legume *L. japonicus*, infection takes place *via* root hair ITs with its cognate symbiont *M. loti*, but plasticity in the infection mode was first revealed in different plant mutants: the *root-hair less 1* and NF-receptor mutants, where intercellular infection was detected at a low frequency (Karas et al., 2005; Madsen et al., 2010). More recently, functional nodules were found to be induced by IRBG74 invading *L. japonicus*, intercellularly. The IRBG74 strain has been isolated from *Sesbania cannabina* nodules and after sequencing assigned as an *Agrobacterium pusense* strain that possesses a symbiotic plasmid. It can infect *Sesbania* spp. either under flooded or non-flooded conditions (Cummings et al., 2009; Mitra et al., 2016). In *L. japonicus* inoculated with IRBG74, the cortical cells are invaded *via* peg-mediated entry from the apoplast into the symbiosome of the infected cells (Figure 1C; Montiel et al., 2021). All these cases of conditional intercellular infection are valuable because they provide the opportunity for direct comparison of the two invasion routes in the same host plants, thus identifying mechanistic commonalities and differences (Goormachtig et al., 2004). Despite this advantage, the molecular components of this mechanism have only been investigated in *S. rostrata* and *L. japonicus*, as described below.

**Figure 2:**
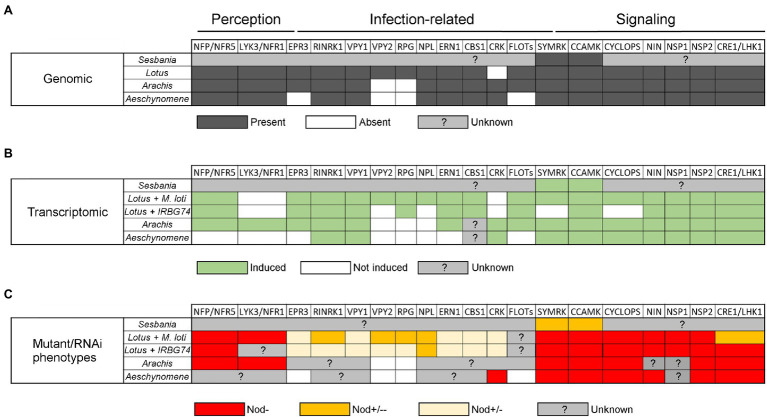
Table comparing data on symbiotic genes involved in different nodulation steps in *Arachis hypogaea*, *Aeschynomene evenia*, *Lotus japonicus*, and *Sesbania rostrata* according to their symbionts. **(A)** Comparison of the presence (dark grey), absence (white), or unknown status (light grey) of symbiotic genes in the genome of *A. hypogaea*, *A. evenia*, *L. japonicus*, and *S. rostrata*. **(B)** Comparison of symbiotic genes that are induced (green), not induced (white) or of unknown status (grey) during nodulation in the four species. For *L. japonicus* this comparison includes the symbiont making intercellular infection (IRBG74) and intracellular infection (*M. loti*). **(C)** Comparison of the phenotypes with different genetic approaches (mutant or RNAi) in the four species and *L. japonicus* with the two symbionts. Red indicates absence of nodules (Nod-), orange indicates an intermediate strong phenotype (Nod+/−−), yellow indicates an intermediate weak phenotype (Nod+/−), and grey indicates the absence of data. For the *Lotus*-IRBG74 interaction, only transcriptome data of early time points is available (3, 5, and 10 dpi).

During the *S. rostrata-A. caulinodans* symbiosis, axillary root-hairs are induced by the presence of the bacteria at the emergence of lateral roots and the intercellular infection process take place at the LRB. The progression of bacteria in the root is followed by a cell death programme in cortical cells that give rise to the formation of an infection pocket (IP; Capoen et al., 2010a). From these structures, rhizobia migrate into inner root cell layers through transcellular ITs or intercellularly. This symbiotic association served as precursor to understanding the molecular components involved in intercellular infection in legumes. One of the first discoveries was to demonstrate that IP formation is a NF-dependent process, since the *A. caulinodans nodA* mutant, disrupted in NF production, is unable to colonize the legume roots (D'haeze et al., 1998). Likewise, in this initial stage, gibberellins, reactive oxygen species and ethylene are produced, playing a positive role (D'haeze et al., 2003; Lievens et al., 2005). Interestingly, intercellular cortical infection was not affected in *S. rostrata* transgenic roots downregulated in the leucine-rich repeat (LRR) receptor-like kinase (SymRK) by RNAi (Capoen et al., 2005). Similarly, RNAi-mediated silencing of the calcium- and calmodulin-dependent protein kinase (CCaMK) did not interfere with the intercellular infection (Capoen et al., 2009). These two players (*SrSymRK* and *SrCCaMK*), crucial for root-hair IT colonization. However, based on the RNAi results they seem not to be necessary for intercellular infection, although they are still required in subsequent stages of the nodulation process (Figure 2). A transcriptomic study on *S. rostrata* roots evidenced 627 differentially-expressed gene tags that are induced during root hair curling and crack-entry rhizobial infection. However, the number of tags was considerably lower during intercellular infection (Capoen et al., 2007). The milder transcriptomic response during intercellular infection indicates that a reduced gene machinery is required in this mechanism. This notion is supported by the less stringent NF recognition in the intercellular symbiotic program compared to the root-hair infection process (Goormachtig et al., 2004).

Similarly, in the *L. japonicus*-IRBG74 association, an IP is formed in the cortical cells after intercellular infection, which follows an inter/intracellular pathway towards the nodule cells (Figure 1C). With the abundant genetic resources in *L. japonicus*, a robust compendium of symbiotic mutants was tested in the interaction with the IRBG74 strain. This approach allowed a core of symbiotic genes involved in the nodulation program to be delimited by the type of rhizobial infection and grouped as preferentially recruited for the intracellular or intercellular colonization in *L. japonicus* (Figure 2). For instance, *nfr5*, *symrk*, *ccamk*, *nin-2*, *nsp1*, and *nsp2* mutants altered in NF perception and the Nod signaling pathway, were unable to induce nodule development in *L. japonicus* after IRBG74 inoculation. Although these findings are relevant, it is still unclear whether intercellular infection occurs in the roots of these mutants, considering the evidence recorded in *S. rostrata*, where intercellular cortical colonization was not prevented in the *SymRK*-RNAi and *CCaMK*-RNAi lines (Capoen et al., 2005, 2009). Unexpectedly, mutants affected in cytokinin-related genes such as *lhk1*, *cyp753a*, and *ipt4* had a more severe negative impact in the intercellular symbiosis with IRBG74. The most prominent difference was the performance of the *lhk1* mutant, since it was unable to form any nodule structure with IRBG74 (Montiel et al., 2021). On the other hand, mutants affected in the infection genes *CYCLOPS*, *CBS*, *EPR3*, or *VPY1* showed comparable symbiotic phenotypes during intra–intercellular interactions (Montiel et al., 2021). However, a milder symbiotic perturbance was observed in the *rbohE*, *rbohG*, *RPG*, *RPG*-Like, *RINRK1*, *ERN1*, and *VPY2* infection gene mutants in the intercellular interaction with IRBG74, compared to the phenotype observed with *M. loti* as the inoculum. These results suggest that an intercellular symbiotic process employs a different, and apparently reduced, repertoire of molecular players compared to intracellular colonization *via* root-hair ITs (Figure 2). This hypothesis is further supported by the remarkable contrast in number of differentially expressed genes (DEGs) in the susceptible zone of roots exposed to *M. loti* or IRBG74; 473 vs. 250, respectively. Interestingly, a large proportion (67%) of the DEGs by *M. loti* were not differentially expressed with IRBG74. Nonetheless, a core of relevant symbiotic genes was upregulated to comparable levels with both symbionts (Montiel et al., 2021).

## Constitutive Intercellular Infection Process

The constitutive intercellular infection process has been reported in certain species of the Mimosoidae-Caesalpinea-Cassieae clade but it is best described in two Papilionoid subclades using different modalities: Genistoids (Direct Entry) and Dalbergioids (Crack Entry; Ibañez et al., 2017). Recently, two legume species from the Dalbergioid subclade, *Arachis hypogaea* (peanut) and *Aeschynomene evenia*, emerged for the study of nodulation (Figure 1A). Since they are phylogenetically related, their comparative analysis is predicted to facilitate the finding of common features related to crack entry. However, *A. evenia* uses a Nod-independent process by interacting with *Bradyrhizobia* lacking the Nod-factors’ nodulation (*nod*) genes. Therefore, studying this singular genetic system is also expected to reveal the minimal set of genes that are required for intercellular infection without the additional components linked to the classic perception of rhizobia. In these two legume species, tufts of multicellular axillary root hairs are present at the base of lateral roots that represent the infection sites. In *A. hypogaea*, the bradyrhizobia pass through the middle lamella between two root-hair cells at the LRB and spread into the cortex *via* the middle lamella. Uptake into the susceptible cell occurs through a structurally-altered cell wall. The Nod factors are not required for colonization of the peanut root surface but are required for the induction of cortical cell division of the first infected cell that will form the nodule primordia, when interacting with its natural symbionts (Ibañez and Fabra, 2011). However, very recent occurrences of Nod-independent nodulation have been reported in *Arachis* when inoculated with non-cognate symbionts, but the underlying mechanisms are not yet documented (Guha et al., 2022). Within the *Aeschynomene* genus, some species nodulate in a NF-dependent fashion and their root infection by bradyrhizobia occurs in a way that is comparable to *A. hypogaea* (Giraud et al., 2007). In contrast, other species such as *A. evenia* nodulate in a Nod-independent way and their root infection is distinctive in that the bradyrhizobia first intensely colonize the axillary root-hairs and then penetrate into the root between axillary root-hair cells or *via* a crack at the lateral root basis (Figure 1B). Another notable feature is that when bacteria progress into the root in the intercellular space, they induce cell collapse among the first-invaded cortical cells, after which they are internalized in an inner susceptible cortical cell by invagination of the cell wall, and the infected cell initiates successive divisions that give birth to the nodule primordia (Bonaldi et al., 2011).

Recent genome sequencing projects and transcriptomic studies have enabled investigation into the presence and expression of symbiotic genes in *A. hypogeae* and *A. evenia*. Almost all the determinants characterized in the model legumes *M. truncatula* and *L. japonicus* are also present in their genome, notably the Nod-factor perception genes *NFP/NFR5* and *LYK3/NFR1*, symbiotic signaling genes *SYMRK*, *POLLUX*, *CCAMK*, *CYCLOPS*, *NIN*, *NSP2*, and *CRE1/LHK1*, and infection-related genes *RINRK1*, *VPY*, *NPL*, *ERN1*, and *CBS1* (Roy et al., 2020; Krönuer and Radutoiu, 2021; Figure 2). In contrast, the *RPG* gene, coding for a coiled-coil protein and linked to infection in *M. truncatula* (Arrighi et al., 2008), is absent from the genome in both of these species (Chaintreuil et al., 2016; Peng et al., 2017; Gully et al., 2018; Karmakar et al., 2019). This suggests that *RPG* is specific to IT-mediated infection and is not involved in intercellular infection. In *A. evenia*, some specificities are also observed with the non-expression of LYK3 and the absence of the infection-related genes *EPR3* and *FLOT*s in the genome (Quilbe et al., 2021). In *A. hypogeae*, known symbiotic genes acting in the common IT mediated nodulation (125 peanut orthologs) are also expressed during symbiosis (Figure 2). The majority of DEGs are observed during nodule functioning, then nodule organogenesis and finally infection (Raul et al., 2022). Transcriptomic analyses of a time-course experiment in *A. evenia* allowed the identification of DEGs at early and late stages of the symbiotic interaction. A small number of DEGs at early stages, corresponding to the colonization of the roots by bradyrhizobia, are linked to responses to stress and abiotic stimuli. In general, the number of DEGs decrease as the nodulation process progresses (Chaintreuil et al., 2016; Peng et al., 2017; Gully et al., 2018; Karmakar et al., 2019). Other gene expression analyses in *A. evenia* have revealed that determinants such as *VPY*, *LIN*, and *EXO70H4*, required for polar growth of infection threads and subsequent intracellular accommodation of symbionts in *M. truncatula,* show a symbiotic expression pattern. However, it is not yet known whether they are involved in intercellular infection and/or only in the latter stages of the symbiotic process (Quilbe et al., 2021).

Genetic analyses have been conducted on these two Dalbergioid species to determine which genes are important for nodulation by intercellular infection (Figure 2). In *A. hypogeae*, a reverse genetic approach based on CRISPR/Cas9 in hairy roots showed that the mutants edited in *AhNFR5* are unable to produce nodules (Shu et al., 2020). In addition, the silencing of three symbiotic genes with RNAi experiments led to the discovery that CCaMK, CYCLOPS and LHK1 are involved in the nodulation process (Sinharoy and DasGupta, 2009; Kundu and DasGupta, 2018; Das et al., 2019). More recently, thanks to map-based cloning and QTL-seq approaches, Peng et al. have shown that the two homoeologs of the *NSP2* gene control nodulation in *A. hypogeae* (Peng et al., 2021). Similarly, in *A. evenia*, some RNAi studies have shown that *SYMRK*, *CCAMK*, and *LHK1* are required for nodulation, although the role of these genes in intercellular infection has not been investigated (Fabre et al., 2015). More recently, in *A. evenia*, a forward genetic approach led to the selection of hundreds of EMS nodulation mutants, among which some Nod^−^ mutants allowed for the identification of *AePOLLUX*, *AeCCAMK*, *AeCYCLOPS*, *AeNIN*, and *AeNSP2* as essential determinants for the establishment of Nod-independent symbiosis. Among all the mutants screened, none of them showed mutations in any LysM-RLK genes, to which NF receptor genes belong. Furthermore, this screen led to the discovery of a novel symbiotic gene, *AeCRK*, coding a Cystein-rich Receptor Kinase that is required to trigger nodulation. The nodule-less phenotype of the *crk* mutants and the expression profile of *AeCRK* suggest a function in both early and later stages of symbiosis. In plants, CRK functions are not clearly established but they are often linked to immunity and the authors suggested that in *A. evenia* this receptor could be involved in ROS signaling during Nod-independent symbiosis (Quilbe et al., 2021). Interestingly, this particular gene is also found in other Papilionoid legumes using intercellular infection, like *Lupin* and *Arachis* spp., but not in those using an IT-mediated infection process, such as *M. truncatula* or *L. japonicus*. Therefore, investigating the role of AeCRK could reveal a novel important function for intercellular infection.

## Conclusion and Future Directions

The evolution of nodulation in legumes and their infection processes has been recently reviewed and explored (Fonseca et al., 2012; Sprent et al., 2017; De Faria et al., 2022), however, since intracellular and intercellular infection occurs in nodulating legume subfamilies that originated nearly simultaneously in evolution (Koenen et al., 2020), it is complicated to determine the ancestral colonization mechanism. Consequently, a more complete understanding of the molecular mechanisms underlying intercellular rhizobial infection in legumes becomes more relevant. Getting a general view is difficult because there are different patterns of intercellular infection and the available molecular data are still fragmented. However, one clear outcome from these studies, the most extensive in *L. japonicus*, is that NF perception and symbiotic signaling are important for intercellular infection (Montiel et al., 2021). *A. evenia* is likely an exception, since there are no indications of any NF receptor involvement in the Nod-independent symbiosis (Quilbe et al., 2021). The next main challenge will be to understand the molecular mechanisms that underpin the passage through the epidermis, the accommodation of bacteria within the apoplast, cell collapse or peg formation, and the entry by cell wall invagination during intercellular infection (Figure 1). They remain largely unknown since they have not been identified with the studies based on knowledge from the IT-mediated infection process.

These mechanisms could be elucidated by screening plant mutants and performing careful microscopic phenotyping for alterations in infection. To perform such an investigation, *L. japonicus* and *A. evenia* appear to be appropriate study models because genetic resources are available for both legumes. The Lotus collection of LORE1 insertion mutants has been mined for genes important for IT-mediated nodulation (Montiel et al., 2021). It can also serve to directly screen mutants involved in intercellular infection. Similarly, the genetic work initiated in *A. evenia*, and that enabled the recent identification of *AeCRK*, can be extended to available infection mutants (Quilbe et al., 2021). Since *L. japonicus* and *A. evenia* belong to distant Papilionoid lineages and make use of different intercellular infection programs, we anticipate that their concomitant study will be complementary to identify novel infection genes. Extending these studies with comparative phylogenomics and transcriptomics studies comparing root-hair IT and intercellular infected legume species would also help define the genetic specificities of intercellular infection. Such knowledge will enrich our understanding of rhizobial infection in legumes. It may also offer new engineering strategies to achieve biological nitrogen fixation in non-legume crops such as cereals.

## Author Contributions

JQ and JM drafted the article and figures. JM, JQ, and JS contributed to conception and design of the work. J-FA and JS contributed to critical revision of the article. All authors contributed to the article and approved the submitted version.

## Funding

This work was supported by the grant Engineering the Nitrogen Symbiosis for Africa made to the University of Cambridge by the Bill & Melinda Gates Foundation (ENSA; OPP11772165), the European Research Council (ERC) under the European Union’s Horizon 2020 research and innovation programme (grant agreement no. 834221), and the project Molecular Mechanisms and Dynamics of Plant-microbe interactions at the Root-Soil Interface (InRoot), supported by the Novo Nordisk Foundation grant no. NNF19SA0059362.

## Conflict of Interest

The authors declare that the research was conducted in the absence of any commercial or financial relationships that could be construed as a potential conflict of interest.

## Publisher’s Note

All claims expressed in this article are solely those of the authors and do not necessarily represent those of their affiliated organizations, or those of the publisher, the editors and the reviewers. Any product that may be evaluated in this article, or claim that may be made by its manufacturer, is not guaranteed or endorsed by the publisher.
